# Land Cover Classification with GF-3 Polarimetric Synthetic Aperture Radar Data by Random Forest Classifier and Fast Super-Pixel Segmentation

**DOI:** 10.3390/s18072014

**Published:** 2018-06-22

**Authors:** Yuyuan Fang, Haiying Zhang, Qin Mao, Zhenfang Li

**Affiliations:** National Key Laboratory of Radar Signal Processing, Xidian University, Xi’an 710071, China; fangyuyuan94@163.com (Y.F.); zhanghaiying4155@163.com (H.Z.); mq199311@163.com (Q.M.)

**Keywords:** GF-3 satellite, polarimetric synthetic aperture radar (PolSAR), land cover classification, Random Forest, super-pixel segmentation

## Abstract

Chinese Gaofen-3 (GF-3), a vital satellite for high-resolution earth observation, was the first C-band polarimetric synthetic aperture radar (SAR) launched in China with a resolution of up to one meter. Polarimetric SAR can obtain the complete physical scattering mechanisms of targets, thereby having the potential to differentiate objects. In this paper, several classification methods are briefly summarized and the types of features that should be chosen during classification are discussed. A pre-classification step is introduced to reduce the workload of precise labeling. The Random Forest classifier, which performs well for many other classification tasks, is used for the initial land cover classification. Then, based on a polarimetric constant false-alarm rate (CFAR) edge detector, a fast super-pixel generation method for polarimetric SAR image is proposed, which does not require the adjustment of parameters in advance. Following that, majority vote is conducted on the initial classification result based on the super-pixels, so that the classification result can be optimized to better meet the mapping requirements. The experimental results based on GF-3 polarimetric SAR data verify the effectiveness of proposed procedure and demonstrate that GF-3 data has excellent performance in land cover classification.

## 1. Introduction

Chinese Gaofen-3 (GF-3), launched August 2016, was the first Chinese C-band polarimetric synthetic aperture radar (SAR). The GF-3 system has high spatial resolution, multi-polarization imaging, and all-weather, all-day observation. The GF-3 system remote sensing product can be used for applications such as marine environment monitoring, resource investigation, and disaster prevention and evaluation [1].

Land cover classification is one of the most important applications in the field of polarimetric SAR research. The classification results can be either directly used in mapping and national land resource statistical research, or as the input for other applications. In the fully polarimetric SAR data, the targets’ structure information can be interpreted as the sum of surface scattering, double-bounce scattering and volume scattering. Surface scattering corresponds to rough surfaces such as bare soil and sand land. Double-bounce scattering relates to dihedral corners such as artificial targets’ ground-wall corners. Volume scattering is associated with random oriented dipoles such as the crown of a tree. As such, polarimetric SAR has the potential to differentiate vegetation, bare land, buildings, water bodies, and so on.

Generally, two methods are available for polarimetric SAR classification: polarimetric-decomposition theorems and statistics-based methods. Even though many classification methods are proposed, they lack of versatility, resulting in dissatisfying classification accuracies [2,3,4,5,6,7].

Polarimetric-decomposition theorems are mainly based on model decomposition or eigenvalue decomposition. Several traditional model-decomposition methods decompose the polarimetric matrix into the sum of several parts, such as Huynen decomposition, Freeman-Durden decomposition, Yamaguchi decomposition, and Cameron decomposition [3,4,5,6,7], which all have good physical interpretability. However, these methods still suffer from many challenges. From the authors’ perspective, the main issues include ambiguity and model mismatch. Ambiguity means one scattering form can be interpreted as many scattering structures, which leads to a large area of false identifications. For example, the crown of trees will cause high-volume scattering proportion, but the rotation of buildings also increases the volume scattering proportion, implying that high-volume scattering can be related to multiple categories. Model mismatch always occurs when the polarimetric data are decomposed with incorrect polarimetric models that do not match the ground objects’ real scattering mechanisms. Therefore, the volume scattering power is overestimated, and negative power will be generated. Sometimes, the polarimetric data of some crops generate puzzling results after Freeman decomposition. Part of the reason for this occurrence is that the leaves and branches of the crops do not match the stochastic volume scattering model. Generally, erroneous estimations mainly exist in the volume scattering models. Therefore, a series of new polarimetric-decomposition methods were proposed, such as Arii decomposition [8] and General Polarimetric Model-Based Decomposition [9]. These methods select the optimal volume scattering model by calculating a constrained optimization problem. In General Polarimetric Model-Based Decomposition, the authors proposed a new concept about the polarization orientation angle (POA) problem so that the polarimetric decomposition is more accurate. However, the task is computationally intensive as the constrained optimization must be computed in each pixel. In conclusion, polarimetric model-decompositions are more similar to feature extraction and interpreting or visual discrimination, but they cannot be directly utilized in classification.

To address these two problems, a hierarchical classification is necessary so that polarimetric-decomposition methods can be used. Since we cannot decompose the polarimetric data of all the ground targets with the same volume scattering model, we could roughly pre-classify the data, then use customized volume scattering models to conduct polarimetric decomposition in each category accordingly. With this method, both problems are considered: through pre-classification, ambiguity can be alleviated to some extent; and through the customized polarimetric volume scattering models, model mismatch will be less likely to occur.

The Wishart classifier is an important statistics-based method in the field of polarimetric SAR classification. Based on the complex Gaussian Circle Distribution, Lee [10] theoretically proved that polarimetric SAR data are Wishart-distributed if the ground target is homogenous and has only one scattering mechanism. To improve upon the Wishart classifier, some non-Gaussian models were proposed which fit the high-resolution polarimetric SAR images [11,12]. Apart from the microwave band and the resolution of the image, one kind of target generally has different scattering mechanisms. That is to say, some ground targets do not meet Wishart distribution in general, such as differently-oriented buildings. Nevertheless, the Wishart classifier is useful in countering changes in incidence angle, which is still worth researching.

With the growing focus on machine learning, an increasing number of machine learning methods have been proposed in the polarimetric SAR image classification field. In 1991, Pottier first used the multi-layer perception neural network (NN) to classify polarimetric SAR images [13]. Then, Support Vector Machine (SVM) [14,15], Extreme Learning Machine (ELM) [16], and Sparse Representation Classifier (SRC) [17] have been studied in polarimetric SAR image classification. Apart from polarization features, some texture features can also be applied to classification. Among those widely used texture features is the gray-level co-occurrence matrix (GLCM), which describes the local information such as mean, variance, contrast, and homogeneity. Compared to the Wishart distribution-based methods, these approaches have the following advantages: (1) they can cope with data that has more complex feature distributions, (2) more representative information can be considered so higher accuracy can be obtained, and (3) compared with the polarimetric decomposition methods mentioned above, the optimization operation is only performed once, resulting in considerable time savings. 

However, the machine learning methods require a certain number of samples and some parameter adjustments. It is difficult to compare the classification accuracy, false alarm rate, and missed alarm rate of different methods precisely due to their different parameter settings. Nevertheless, fast classification speed is desirable because parameter adjustment is usually conducted several times. In conclusion, faster speed, higher accuracy, and less parameter adjustment are essential elements in choosing the classifier and optimizing the result.

The Random Forest (RF) is an ensemble learning technique that has been receiving increasing attention in many Kaggle competitions and in satellite image classification [18,19]. Based on a series of random generated decision trees, RF assigns a specific class by the ensemble vote of these trees. RF has the following advantages [20]: (1) higher classification accuracy than SVM and other prevailing classifiers; (2) low bias, variance, and generalization error, reducing the likelihood of over-fitting; (3) fast learning process with low memory cost; (4) for unbalanced classification data sets, RF can balance the error; and (5) as only one to three parameters will be adjusted, RF is easy to handle and adjust.

The common drawback of these methods is that the classification process is based on pixels. Due to the speckle noise, error classification often occurs. In addition, according to the mapping requirement, some isolated ground objects should be ignored. Also, generally, neighbor pixels tend to belong to the same category. Several research works have focused on smoothing the classification result to obtain higher accuracy. Markov random field (MRF) has been introduced [21,22], using Wishart probability density to construct energy models. Other methods are based on super-pixel generation and majority vote. A super-pixel is defined as a homogeneous area. Ersahin proposed a method based on Graph Theory using Normalized Cut Algorithm to complete PolSAR image segmentation [16], but the memory and time cost were considerable, which detracted from its practicality. Qin applied a simple linear iterative clustering (SLIC) method for super-pixel generation in polarimetric SAR images [23], which is both faster and performs well. However, parameter adjustment is inevitable with these methods. To obtain a good segmentation result, a series of parameters must be tested, and the algorithm runs repeatedly, leading to a considerable time cost.

In this paper, a practical and fast polarimetric SAR classification procedure is proposed. The procedure is validated using GF-3 polarimetric SAR images. The rest of the paper is organized as follows. In Section 2, the basics of proposed classification process are presented. Section 3 provides the experimental results based on GF-3 data and the related discussions. Conclusions and perspectives of future GF-3 classification products are outlined in Section 4.

## 2. Methodology

### 2.1. Basics of Polarimetric SAR

For polarimetric SAR, under the reciprocity assumption $S_{HV}=S_{VH}$, the full polarimetric information can be represented in the form of the scatter vector [2] formulated as:

(1) $$k=\frac{1}{\sqrt{2}}{\left[S_{HH}+\;S_{VV}\;,\;S_{HH}-S_{VV}\;,\;\sqrt{2}S_{HV}\right]}^{T}$$

where $S_{HV}$ is the echo transmitted in horizontal polarization and received in vertical polarization, $S_{HH}$ is the echo transmitted in the horizontal polarization and received in the horizontal polarization, and $S_{VV}$ is defined similarly for vertical polarization. 

The *n*-look coherency matrix *T* without POA compensation is defined as:

(2) $$T=\frac{1}{n}\sum_{i=1}^{n}k_{i}{k_{i}}^{H}=\left[\begin{array}{l} T_{11}\;T_{12}\;T_{13}\\ T_{21}\;T_{22}\;T_{23}\\ T_{31}\;T_{32}\;T_{33} \end{array}\right]$$

The scattering power of all polarization channels is:

(3) $$span=T_{11}+T_{22}+T_{33}$$

The coherency matrix *T* [24] with POA compensation by angle *θ* is formulated as:

(4) $$T(\theta )=R_{3}(\theta )\cdot T\cdot R_{3}^{H}(\theta )$$

where the rotation matrix $R_{3}(\theta )$ is:

(5) $$R_{3}(\theta )=\left[\begin{array}{l} 1\;0\;0\\ 0\;\cos2\theta \;\sin2\theta \\ 0\;-\sin2\theta \;\cos2\theta \end{array}\right]$$

The angle *θ* is the equivalent rotation angle of the target along the antenna. Usually, *θ* is estimated through a geometric or cross-polarization term $T_{33}$ [25,26].

Cloude and Pottier [2] proposed an eigenvalue decomposition method that extracts the following features from the matrix *T*: entropy *H*, anisotropy *A*, and the mean alpha angle $\underline{\alpha }$. Based on Cloude decomposition, Van Zyl proposed the radar vegetation index (RVI) [27]. These features are defined as follows:

(6) $$\left\{ \begin{array}{l} H=-\sum_{i=1}^{3}p_{i}\log_{3}(p_{i}),p_{i}=\frac{\lambda_{i}}{\sum_{k=1}^{3}\lambda_{k}}\\ A=\frac{\lambda_{2}-\lambda_{3}}{\lambda_{2}+\lambda_{3}},\\ \underline{\alpha }=\sum_{i=1}^{3}p_{i}\alpha_{i}\\ RVI=\frac{4\lambda_{3}}{\lambda_{1}+\lambda_{2}+\lambda_{3}} \end{array}\right.$$

where $\lambda_{i}(i=1,2,3)$ are the eigenvalues of *T*. In addition, $\alpha_{i}(i=1,2,3)$ in the equations, related to scattering mechanism, are extracted from the eigenvectors of *T*.

As Si-Wei Chen suggests [9], the general form of polarimetric model-based decomposition is

(7) $$T=f_{vol}\cdot \langle T_{vol}\rangle +f_{db}\cdot \langle T_{db}(\theta_{db})\rangle +f_{odd}\cdot \langle T_{odd}(\theta_{odd})\rangle +f_{hel}\cdot \langle T_{hel}\rangle +\ldots +T_{residual}$$

where $T_{db}(\theta_{db})$ denotes the double-bounce scattering model rotated by angle $\theta_{db}$ and $f_{db}$ represents the coefficient of the double-bounce scattering component. The volume scattering model, surface scattering model, and helix scattering model are similarly defined. Equation (7) indicates that different kinds of ground objects, especially crops, have different volume scattering models. In Equation (7), 〈*〉 means choosing the exact or the most fitted model, and $T_{residual}$ denotes the residual matrix.

In traditional model-based classification, useful and representative information for classification includes *span* and the ratio of $f_{vol}$, $f_{db}$, and $f_{odd}$. The hidden information includes the type of $T_{vol}$, and some reflection coefficients such as α and β. However, the precise estimations of the type of $T_{vol}$, α, and β are computationally expensive. Notably, the polarimetric decomposition process is similar to linear dimensionality reduction. If *span*, $f_{vol}$, $f_{db}$, and $f_{odd}$ can differentiate ground objects, then the original elements in *T* can also differentiate these ground objects.

### 2.2. Random Forest

Random Forest (RF) is an ensemble learning algorithm that integrates multiple classifiers. Its basic unit is a decision tree. RF is a powerful classification tool with the unbiased estimation of the classification result. Cross-verification is virtually not required because RF can be evaluated internally during the training. For ground objects of the same type but with differently distributed features, RF can directly classify the objects into one class. The detailed steps of the RF algorithm are described in Surhone et al. [28].

### 2.3. Feature Selection

Feature selection is quite important because each feature should be related to some categories. In the polarimetric SAR classification field, the physical meaning of each feature is easy to determine due to the existence of the classical models. In our proposed method, the selected features and the physical meanings are provided in Table 1.

The following are some explanations about feature selection. First, all the features should be scaled to 0–1. In Table 1, span1 is the normalized span; $\underline{\alpha }$, which ranges from 0 to 90, should also be linearly scaled to 0–1. $T_{33}/span$, which is the feature representing the volume scattering characteristic, is not considered here because it is highly linear with $T_{11}/span$ and $T_{22}/span$. Natural ground objects generally meet the assumption that $\langle S_{HV}S_{HH}^{*}\rangle \approx \langle S_{HV}S_{VV}^{*}\rangle \approx 0$, whereas oriented artificial objects do not meet this assumption. Therefore $\left|T_{23}\right|/\sqrt{T_{22}\cdot T_{33}}$, which equals the phase of $\langle (S_{HH}-S_{VV})S_{HV}^{*}\rangle$, is also effective at distinguishing artificial objects [29].

Some other parameters in Equation (7) are not considered here because they are also highly linear with the elements in *T* as analyzed above. For example, the variable $P_{v}$ in Freeman-Durden decomposition actually equals $4T_{33}$ [24].

Even though nearly all the information in *T* has been used, *H*, *A*, $\underline{\alpha }$ and *RVI* are still recommended because their extractions are not a linear dimensionality reduction process. Additionally, these parameters all have physical explanations, indicating they are good features in the classification. Generally, these parameters are more effective in crop classification [30] than the parameters in Equation (7). Therefore, adding these features will reduce the nonlinear requirements for classifiers. 

Information redundancy exists in those selected features. The selected features have almost obtained all the information in the polarimetric matrix *T*. As the classification is based on pixels, obtaining tens of thousands of samples is easy. The greater the number of samples, the stronger the ability of the classifier to filter the features. Therefore, if extra features such as $\left|T_{12}\right|/span,\left|T_{23}\right|/span$ and $\left|T_{13}\right|/span$ are used, they do not need to be related to any category. Additionally, so does the addition of $\left|T_{12}\right|/\sqrt{T_{11}\cdot T_{22}}$ and $\left|T_{13}\right|/\sqrt{T_{11}\cdot T_{33}}$, which are weak in theoretical support. 

GLCM features provide texture information, which are not recommended to be added into the first training because the performance of these features is not stable on different images. They are very computationally intensive and parameter adjustment is inevitable in their extraction. A better approach is to train the classifier without GLCM features for the first time, with the aim of validating the effect of training sample selection. Besides, the parameters of GLCM features should be tuned with a small set of oriented building samples. Then the features can be added in the following training.

### 2.4. Fast Super-Pixel Segmentation Algorithm

Although other super-pixel segmentation methods are available [21,22,23], their common problem is the need to pre-set the parameters, and setting the best parameters is challenging. The proposed segmentation method has almost no need to set the parameters in advance. To be specific, this method calculates the edge map through a CFAR edge detector [31], filters out the noise by setting a threshold, and then a watershed algorithm is used to complete segmentation. Compared to other methods, this method has a fast segmentation speed and low parameter adjustment cost.

#### 2.4.1. Dissimilarity of Two Regions

If the scattering characteristics of two adjacent areas are different to a certain degree, a line of edge in the middle may exist. Conradsen et al. [32] utilized the Wishart test statistic to measure the pairwise dissimilarities, which formulated a likelihood-ratio function to present the distance of two regions. Suppose many regions exist, for pixels in the *i*th and *j*th regions, i.e., for the matrices sets $R_{i}=\{T_{1}^{i},T_{2}^{i},\ldots ,T_{N_{i}}^{i}\}$ and $R_{j}=\{T_{1}^{j},T_{2}^{j},\ldots ,T_{N_{j}}^{j}\}$, the hypothesis test is:

(8) $$\left\{ \begin{array}{l} H_{0}:{\hat{\Sigma }}_{i}=\;{\hat{\Sigma }}_{j}\\ H_{1}:{\hat{\Sigma }}_{i}\neq {\hat{\Sigma }}_{j} \end{array}\right.$$

where ${\hat{\Sigma }}_{i}$ and ${\hat{\Sigma }}_{j}$ denote the maximum likelihood Wishart center of the *i*th and *j*th regions, respectively, i.e., ${\hat{\Sigma }}_{i}=\frac{1}{N_{i}}\sum_{n=1}^{N_{i}}{Τ}_{n}^{i}$ and ${\hat{\Sigma }}_{j}=\frac{1}{N_{j}}\sum_{j=1}^{N_{j}}{Τ}_{n}^{j}$; and $N_{i}$ $N_{j}$ are the number of pixels in the *i*th region and *j*th region, respectively. Hypothesis $H_{0}$ states that the *i*th region and the *j*th region are homogeneous against the alternative hypothesis $H_{1}$, which states that the *i*th region and the *j*th region are different areas. Notably, $H_{0}$ is a null hypothesis. However, if the distance between ${\hat{\Sigma }}_{i}$ and ${\hat{\Sigma }}_{j}$ is quite small, we assume that $H_{0}$ will be accepted; otherwise $H_{0}$ will be rejected.

If the *i*th region and the *j*th region are homogeneous, the conditional probability density function (PDF) is:

(9) $$p(R_{i},R_{j}|\hat{\Sigma })=\prod_{n=1}^{N_{i}+N_{j}}p({Τ}_{n}|\hat{\Sigma })$$

where $\hat{\Sigma }=\frac{1}{N_{i}+N_{j}}\sum_{n=1}^{N_{i}+N_{j}}{Τ}_{n}$. ${Τ}_{n}$ is the matrix *T* of the *n*th point in the both the *i*th and the *j*th regions.

Otherwise, if the *i*th region and the *j*th region are different regions, the conditional PDF is:

(10) $$p(R_{i},R_{j}|{\hat{\Sigma }}_{i},{\hat{\Sigma }}_{j})=p(R_{i}|{\hat{\Sigma }}_{i})p(R_{j}|{\hat{\Sigma }}_{j})=\prod_{n=1}^{N_{i}}p({Τ}_{n}^{i}|{\hat{\Sigma }}_{i})\prod_{m=1}^{N_{j}}p({Τ}_{m}^{j}|{\hat{\Sigma }}_{j})$$

The pairwise distance between the *i*th and *j*th regions can be derived from a test statistic such as the likelihood ratio test. Based on Wishart distribution, the distance could be calculated in the form of:

(11) $$Q=\frac{p(H_{1})}{p(H_{0})}=\frac{p(R_{i},R_{j}|{\hat{\Sigma }}_{i},{\hat{\Sigma }}_{j})}{p(R_{i},R_{j}|\hat{\Sigma })}=\frac{{\left|{\hat{\Sigma }}_{i}\right|}^{LN_{i}}{\left|{\hat{\Sigma }}_{j}\right|}^{LN_{j}}}{{\left|\hat{\Sigma }\right|}^{L(N_{i}+N_{j})}}$$

where L indicates the number of looks.

Then, then the distance is defined as:

(12) $$\begin{array}{ll} D_{S}(R_{i},R_{j}) & =-\frac{1}{L}\ln Q\\ & =(N_{i}+N_{j})\ln\left|\hat{\Sigma }\right|-N_{i}\ln\left|{\hat{\Sigma }}_{i}\right|-N_{j}\ln\left|{\hat{\Sigma }}_{i}\right| \end{array}$$

#### 2.4.2. Super-Pixel Generation

**Figure 1:**
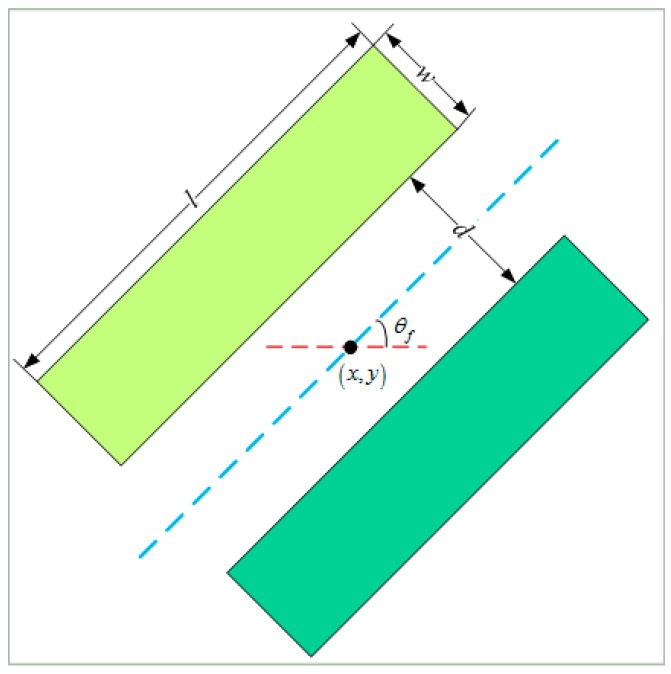
Oriented rectangular filter and its parameter configuration.

Inspired by previous studies [31,33], the edge map was calculated through a set of oriented pairwise rectangular filters, and an example is shown in Figure 1. Generally, the parameter configuration of the oriented filter is $\left\{l,w,d,\theta_{f}\right\}$, where l denotes the filter’s length, w indicates its width, d is distance between two rectangles, and $\theta_{f}$ is the set of orientations of the filter, which are marked in Figure 1. In this paper, {l,w,d}={7,4,1} with the unit of pixel, and $\theta_{f}=\{\frac{\pi }{8},\frac{2\pi }{8},\ldots \frac{8\pi }{8}\}$.

For pixel (x,y), according to Equation (12) and considering that $N_{i}=N_{j}$, the distance of the two rectangular regions in direction $\theta_{f}$ can be formulated as:

(13) $$D_{w}(x,y,\theta_{f})\;=2\ln\left|\hat{\Sigma }(x,y,\theta_{f})\right|-\ln\left|{\hat{\Sigma }}_{1}(x,y,\theta_{f})\right|-\ln\left|{\hat{\Sigma }}_{2}(x,y,\theta_{f})\right|$$

where ${\hat{\Sigma }}_{1}(x,y,\theta_{f})$ and ${\hat{\Sigma }}_{2}(x,y,\theta_{f})$ denote the max-likelihood *T* centers of two square regions in direction $\theta_{f}$ at the point (x,y), respectively. $\hat{\Sigma }(x,y,\theta_{f})$ denotes the max-likelihood *T* center of the merging area of the two square regions.

To scale the range into 0–1, the distance can be mapped as:

(14) $$rF\left(x,y,\theta_{f}\right)\;=\;\frac{1}{1+D_{w}\left(x,y,\theta_{f}\right)}$$

Then the edge similarity is defined as:

(15) $$e(x,y)=1-\underset{\theta_{f}}{\min}(rF\left(x,y,\theta_{f}\right)),f=1,2,\ldots 8$$

As discussed, if pixel (x,y) is on a homogeneous area, the elements of $\left\{D_{w}(x,y,\theta_{f})|f=1,2,3\ldots 8\right\}$ will all be close to 0, so that e(x,y) will be close to 0. On the contrary, if pixel (x,y) is on the edge, some elements of $\left\{D_{w}(x,y,\theta_{f})|f=1,2,3\ldots 8\right\}$ will be large, which indicates that e(x,y) will be closer to 1. To conclude, e(x,y) represents the likelihood of the pixel being on the edge.

However, in a homogeneous area, e(x,y), though close to 0, still fluctuates to some extent. To prevent over-segmentation in the subsequent processing, a parameter λ(0<λ<1) was introduced in Equation (15) to suppress these fluctuations. In most cases, λ is the only parameter to set and adjust. The suppression is formulated as:

(16) $$eF(x,y)\;=\;\left\{ \begin{array}{ll} e(x,y), & g(x,y)\geq \lambda \\ 0, & others \end{array}\right.$$

Then the watershed algorithm is used on the result of Equation (16) to obtain the segmentation result. Using this method, we obtain the super-pixels with a boundary. Then, the boundary can be removed by dilate operation.

In addition, if Wishart distribution is not satisfied in some high-resolution polarimetric SAR images acquired from other sensors, the distance of two pairwise rectangles should be modified. In that case, based on polarization contrast enhancement, the $rF\left(x,y,\theta_{f}\right)$ in Equation (14) can be calculated as:

(17) $$rF\left(x,y,\theta_{f}\right)\;=\;\underset{\left|k\right|=1}{\min}(\frac{k^{H}{\hat{\Sigma }}_{1}(x,y,\theta_{f})k}{k^{H}{\hat{\Sigma }}_{2}(x,y,\theta_{f})k},\frac{k^{H}{\hat{\Sigma }}_{2}(x,y,\theta_{f})k}{k^{H}{\hat{\Sigma }}_{1}(x,y,\theta_{f})k})$$

The solution of k is a generalized Rayleigh quotient problem. Therefore, Equation (17) can be calculated as:

(18) $$rF\left(x,y,\theta_{f}\right)\;=\;\min(\lambda_{\min}({\hat{\Sigma }}_{2}{\left(x,y,\theta_{f}\right)}^{-1}{\hat{\Sigma }}_{1}(x,y,\theta_{f})),\lambda_{\min}({\hat{\Sigma }}_{1}{\left(x,y,\theta_{f}\right)}^{-1}{\hat{\Sigma }}_{2}(x,y,\theta_{f})))$$

where $\lambda_{\min}(*)$ is the operation used to take the minimum eigenvalue of the matrix.

#### 2.4.3. Acceleration

The calculation of the Wishart distance is computationally slow due to the multiple iterations required to conduct matrix operations. To accelerate the process, some parallelization or vectorization techniques can be considered. As shown in Equation (13), all the calculations involve the determinant of the third-order matrix, which is a fixed operation. Thus, the third-order matrices should be stacked at the beginning, and then the parallel determinant operation should be conducted, which considerably shorten the computation time.

#### 2.4.4. Majority Vote

**Figure 2:**
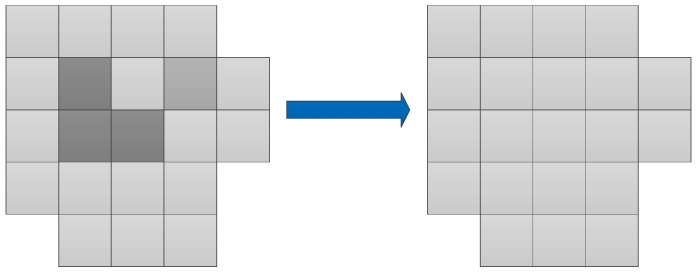
The majority vote process.

The principle of majority voting is to count the proportion of different points in a super-pixel, and then select the category that has the highest frequency to represent the entire super-pixel. Figure 2 outlines the majority vote process. The entire block is a demonstration of one super-pixel. In the block, different colors represent different categories.

#### 2.4.5. Algorithm and Time Analysis

In summary, the whole segmentation algorithm is shown in Algorithm 1. The whole classification procedure is presented in Figure 2.

**Algorithm 1:** Algorithmic steps of Fast Super-Pixel Generation**Input:***T_11_,T_12_,T_13_,T_21_,T_22_,T_23_,T_31_,T_32_,T_33_* of the whole PolSAR image; threshold λ(0<λ<1);$\left\{l,w,d,\theta_{f}\right\}$; **Steps:**$\mathrm{Set}\;\{l,w,d,\theta_{f}\}$ as the configuration of the oriented rectangular filters;For each $\theta_{f}$, use the oriented rectangular filters to compute $\hat{\Sigma }(x,y,\theta_{f})$,${\hat{\Sigma }}_{1}(x,y,\theta_{f})$, and ${\hat{\Sigma }}_{2}(x,y,\theta_{f})$;For each $\theta_{f}$, use the parallel technique to compute Equation (13);Calculate Equations (14) and (15) to determine edge similarity;Calculate Equation (16) through λ;Use the watershed algorithm on the result of step 5 to obtain the segmentation result; Use the dilate operation on the result of step 6 to remove the boundary of the region.
**Result:** Super-pixels without boundary.

When λ is changed, the only time cost occurs with steps 5, 6, and 7, which are generally accepted as the fast algorithms. 

## 3. Experimental Results

### 3.1. Experimental Data

**Figure 3:**
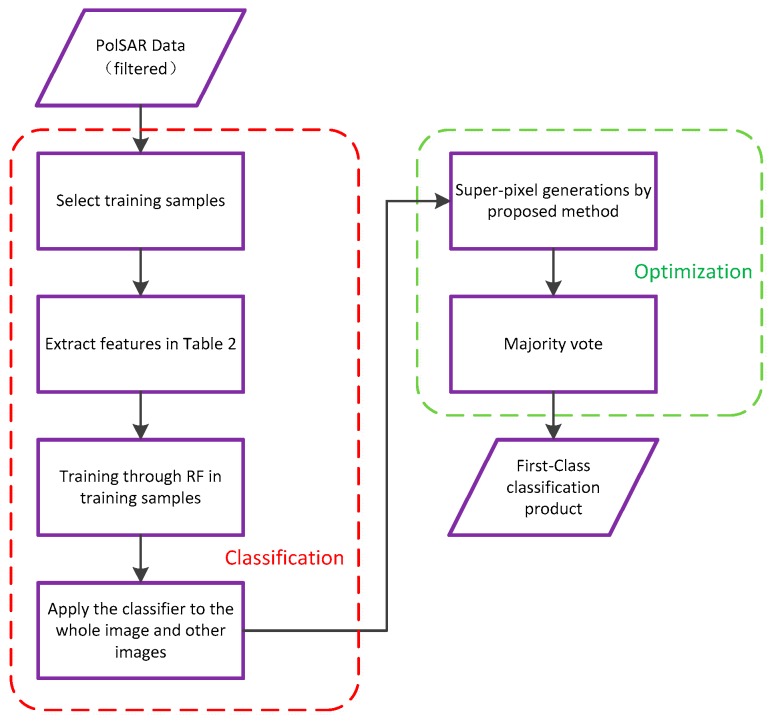
The proposed classification procedure on Chinese Gaofen-3 (GF-3).

The polarimetric SAR data sampled in San Francisco by GF-3 was chosen for this experiment, which can be downloaded from https://www.ietr.fr/GF3/. The Pauli decomposition image is shown in Figure 3a. This region has many types of land resources, including sea water, buildings with different orientations, vegetation, bare soil, and grass land. The used GF-3 polarimetric SAR data were acquired on 15 September 2017 on an ascending pass with right looking direction and its pixel space was about 5.37 × 2.25 m^2^. The image size was 4500 × 3500 pixels and the data were filtered by a 5 × 5 refined Lee filter.

**Figure 4:**
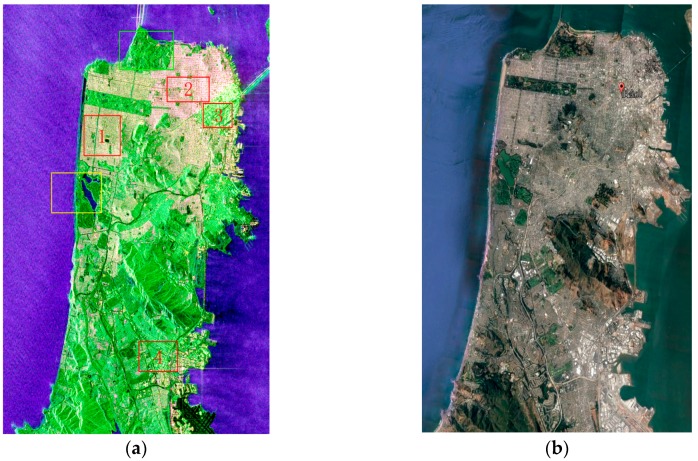
(**a**) The Pauli-basis images of GF-3 data sampled on 15 September 2017; (**b**) Google Earth images sampled on 2 September 2017. In (**a**), the areas inside the red boxes 1, 2, 3, and 4 represent buildings with different scattering characteristics. The areas in the green and yellow boxes are analyzed in Figure 5.

**Figure 5:**
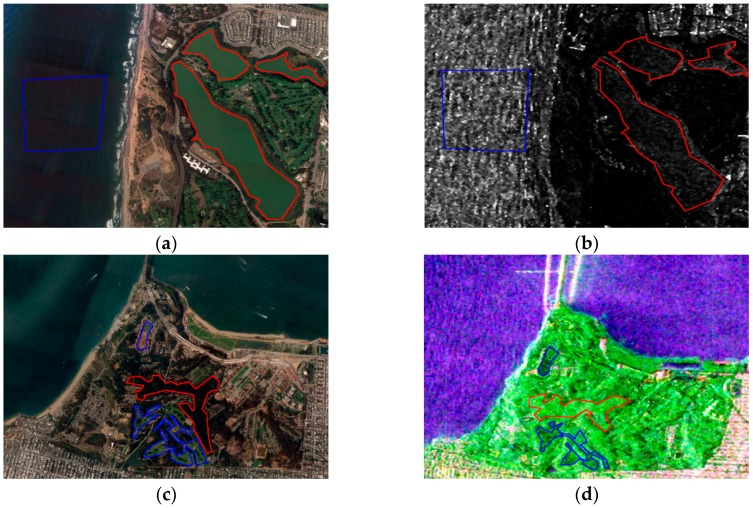
(**a**) Google Earth image of the area marked with the yellow boundary in Figure 4a; (**b**) corresponding span of polarimetric synthetic aperture radar (SAR) image; (**c**) Google Earth image of the area marked with a green boundary in Figure 4a; and (**d**) related polarimetric SAR image.

The following provides a brief introduction about the land resources in the map. Scattering mechanisms of buildings vary along different azimuths, as demonstrated in the red boxes labeled 1, 2, 3, and 4 in Figure 4a. In Figure 5a,b, the ground type in the red polygon is a lake, whereas the ground type in the blue polygon is the sea. Due to the dielectric constant of seawater and the existence of waves, the surface scattering intensity of seawater is higher than that of the lake, indicating that the sampling of water should include both samples. In Figure 5c,d, the ground type in the region with the red boundary is vegetation, whereas the area with blue boundary represents bare soil with vegetation. According to the survey, the main types of vegetation include coniferous forests, such as poplars and pine trees. Though trees and soils all demonstrate high-volume scattering, Figure 5c,d state that the intensity of trees is higher than that of grass and bare soil, which is consistent with common sense.

The experiment and analysis is arranged was follows. First, we adopted a preprocessing step to label the map precisely. In the next step, we compared classification and conducted super-pixel generation. Finally, the result was determined and evaluated.

### 3.2. Sampling

According to the optical image, we inferred that this area mainly included the categories of Artificial architecture, Water body, Tree, Smooth road, and Soil. Figure 5d demonstrates those classes with corresponding labeling colors. Soil includes bare soil and grass land. As Figure 5c,d demonstrate, labeling the polarimetric image is much harder than labeling the optical images. Labeling the samples to the image pixel by pixel would be a laborious project to ensure the correctness of samples.

**Figure 6:**
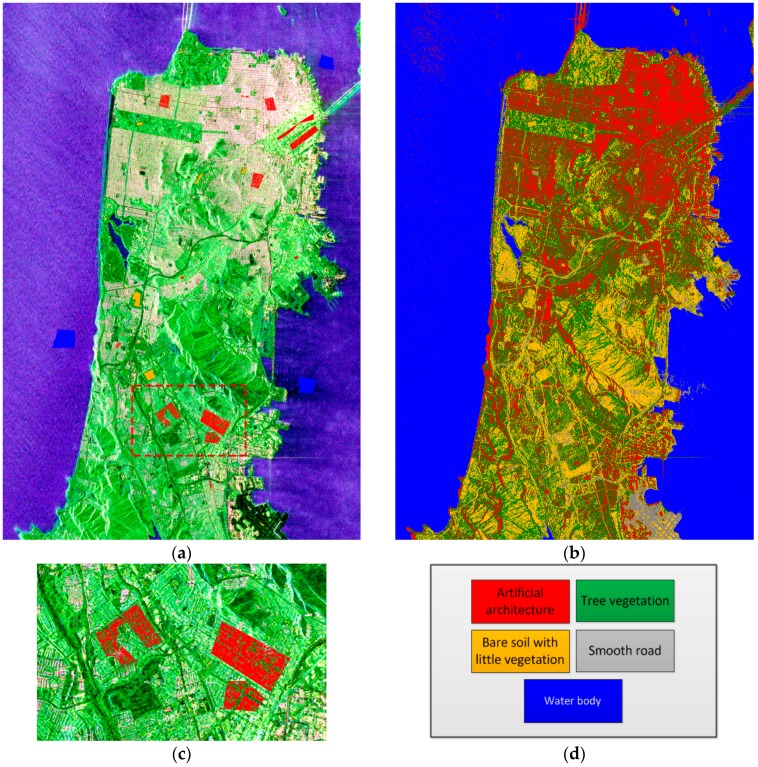
(**a**) Roughly selected samples; (**b**) The result obtained using the Fast Wishart classifier without iteration; (**c**) The area with red boundary in (a), which shows how to eliminate the incorrect samples by setting a threshold to span; (**d**) The label colors of the five categories.

Though the polarimetric data still meets Wishart distribution in this image, some categories may have various scattering mechanisms, indicating that the Wishart classifier was not the best choice in this case. However, the Wishart classifier is insensitive to outliers to some extent. Therefore, pre-classifying the image using the Wishart classifier with rough sampling is recommended. As such, we obtain a primary and intuitive analysis of the data characteristics, and the number of classification categories can be determined to some extent. Additionally, rough sampling can be quite fast so as to reduce the workload for precise labeling. Li et al. proposed the Fast Wishart classifier [34], which is tremendously faster with the same result generated using the Wishart classifier. Figure 6 demonstrates how to roughly select samples with polygons and exhibits the result obtained via the Fast Wishart classifier. Among those labels, the Buildings class is not homogeneous in the polygon. Therefore, a threshold to *span* was set to eliminate those points that are not the roof of the buildings, as shown in Figure 6c.

**Figure 7:**
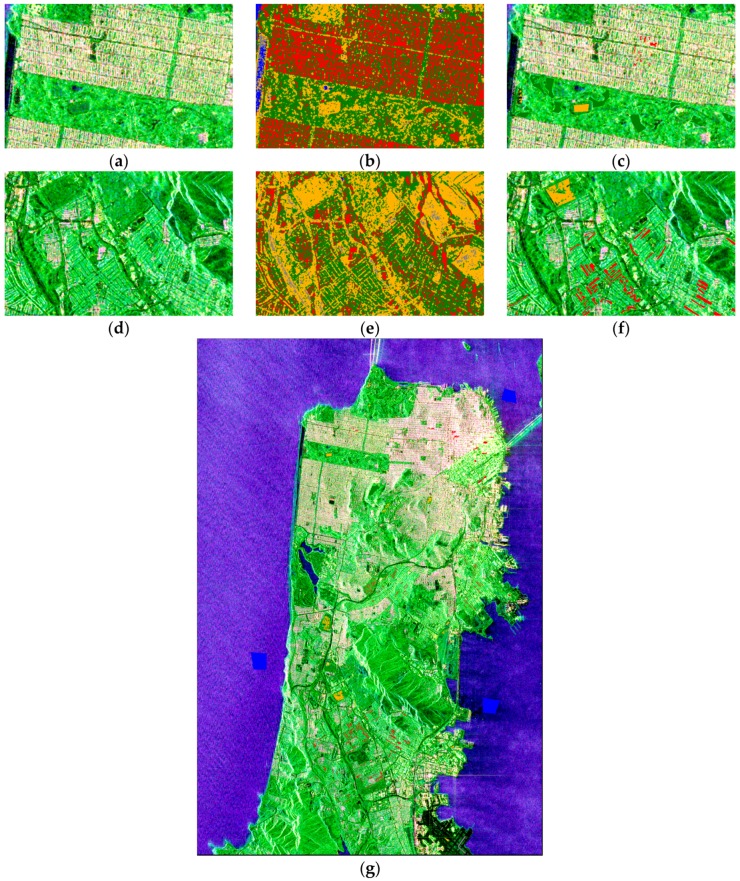
(**a**) The Pauli-basis image of Golden Gate Park; (**b**) pre-classification result; and (**c**) precise labeling; (**d**) The Pauli-basis image of the area in the red box from Figure 6a; (**e**) the corresponding pre-classification result; (**f**) the corresponding precise labeling; and (**g**) the precise labeling of the whole image.

The following provides a discussion about the result displayed in Figure 6b. The image size is 4500 × 3500 pixels, whereas the classification time was only 2.77 s, which is impressive given the number of classes on the map. According to the optical image in Figure 4b, the result misclassifies large areas of buildings in the south. Figure 7 demonstrates how to precisely label the polarimetric map. Comparing Figure 7a and Figure 7b, the buildings, trees, and soil in Figure 7b are more distinguishable. Therefore, they can be more accurately labeled according to Figure 7b; Figure 7c exhibits that process. Figure 7e highlights a large area of misclassifications, but the distinction of the roofs and the land has become clearer. Part of the misclassified points are marked with the right labels referring to the optical image, as shown in Figure 7f. Based on the pre-classification result, label marking will be more accurate, and the isolated misclassified labels can be eliminated from the polygons in an easier way.

### 3.3. Classification Comparison

**Figure 8:**
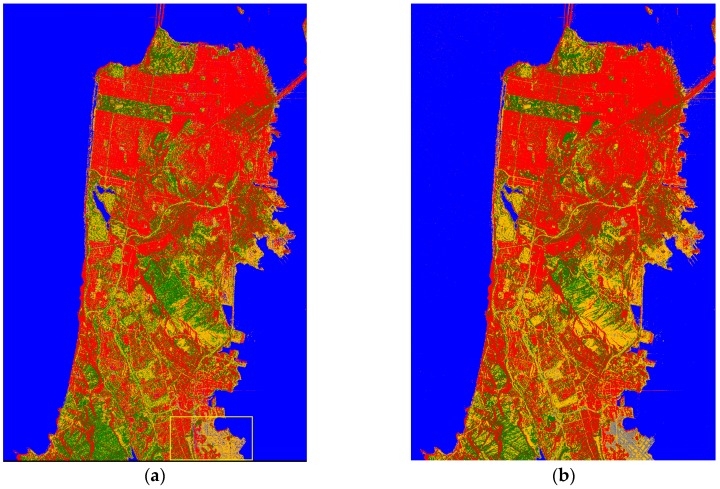
The classification result obtained with (**a**) the Extreme Learning Machine (ELM) and (**b**) Random Forest (RF). The area with yellow boundary in Figure 8a has obvious misclassifications.

Some tests were conducted to compare ELM [16] and RF. Figure 8 demonstrates the classification results. The sample was randomly split into 70% training set and 30% testing set. The comparison between ELM and RF in terms of training accuracy, testing accuracy, training time, and classification time is shown in Table 2.

The yellow box in Figure 8a shows that the runway at the airport was misclassified in a large area, whereas the same area in Figure 8b exhibits the correct classification. The reason for this is that the total number of runway samples was relatively small, as shown in Table 2B. This comparison verifies the characteristics of RF mentioned in Section 1: RF can cope with unbalanced samples to some extent. Though comparison results change with the parameter, both classification results and the indicators in Table 2 both demonstrate RF’s superiority.

Table 2C,D demonstrate the confusion matrices of ELM and RF, respectively. The results were calculated in the testing set of the selected samples. As shown in the tables, RF performed better than ELM in almost every measure. Table 2D also infers that the Tree and Building classes are the hardest to distinguish, whereas very few misclassified points occurred in the other classes and these two classes. Considering that RF is such a powerful tool, some other methods-based merely on physical scattering mechanisms would also encounter difficulties with these points in the polarimetric image acquired from GF-3.

### 3.4. Super-Pixel Generation Experiments

**Figure 9:**
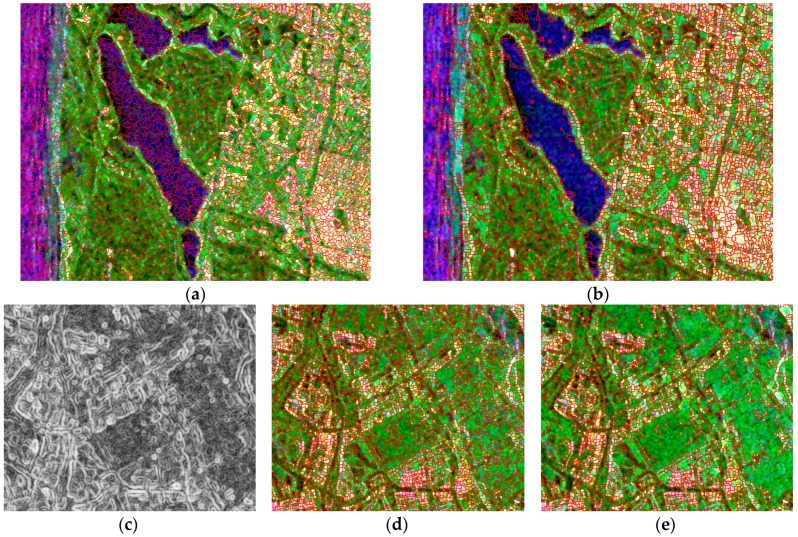
Part of the segmentation result, where the red line presents the boundary. (**a**) The result obtained by simple linear iterative clustering (SLIC), with desired super-pixel number k=135,000, compactness m=0.1, and 10 iterations; (**b**) The result obtained used our proposed method in the same region with the parameter λ=0.8; (**c**) Part of the edge mapping values obtained with Equation (15); (**d**) The segmentation result of the same region with; (**e**) The segmentation result with λ=0.8.

To meet the mapping demand, locally isolated points should be smoothed and ignored. When the classifier is generalized to the whole image, misclassifications still occur, which may be caused by speckle noise and sample selection bias. The contextual information improves the classification. Part of the result of the proposed super-pixel generation is demonstrated in Figure 9. Figure 9a,b exhibit the different effects of SLIC [23] and fast super-pixel segmentation. Figure 9c depicts part of the edge map calculated by Equation (15). Figure 9d,e are the super-pixel generation results with λ=0.7 and λ=0.8, respectively, which demonstrate the process of adjusting λ to control the size of the super-pixels. The entire running time and parameter adjustment time are shown in Table 3.

As shown in Table 3, SLIC needs to pre-set parameters, therefore its adjusting time is equal to the running time, indicating that a long time is required to obtain the best segmentation result. Similarly, the methods previously reported [21,22,35,36] have the same deficiency. Figure 9c shows that the boundary of the super-pixels fits the edge of the image textures because the super-pixel generation is based on edge extraction. 

Adjustment in super-pixel generation is important. The only parameter λ in the proposed method controls the size of the super-pixels as shown in Figure 9d,e. As λ increases, the super-pixel size increases. If the super-pixel is too small, some local noise likely remains as the main local component. The parameter was adjusted several times in this experiment, and λ=0.73 was chosen as the final threshold.

### 3.5. Final Result and Evalution

**Figure 10:**
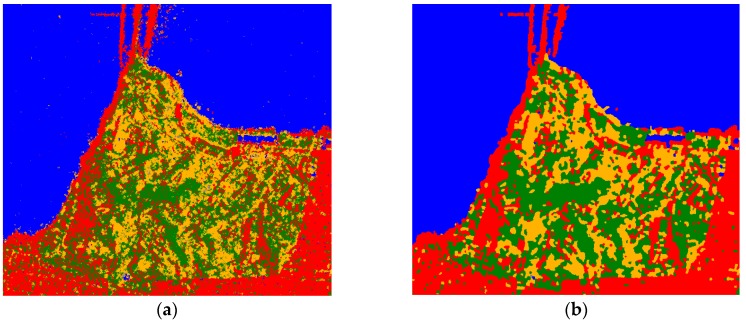

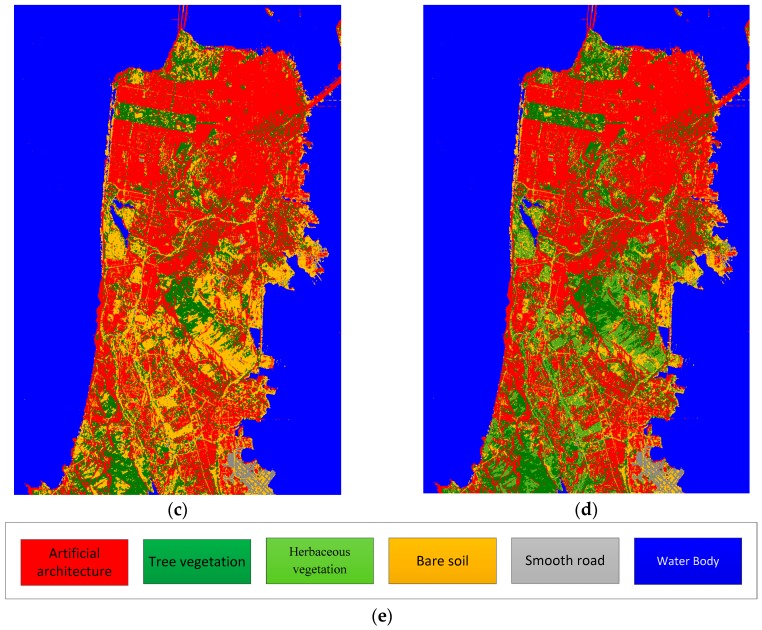
(**a**) Part of RF classification result; (**b**) corresponding area after majority vote; (**c**) whole classification result after majority vote; (**d**) updated result for the “Bare soil with little vegetation” class, divided into Bare soil and Herbaceous vegetation classes; and (**e**) the colormap for each category.

Figure 10a,b demonstrate how the local misclassifications are eliminated by majority vote. Figure 10c shows the whole optimized classification result obtained with RF. The class labeled in brown is a combination of bare soil and grassland. These classes are hard to distinguish with the Pauli-basis when we used samples from this data. As we suggested, when a hierarchical classification is formed, the false alarm rate and the missed alarm rate in model-based decomposition decrease. Figure 10d displays the updated classification result using Freeman decomposition only for the Bare soil with little vegetation class. After the POA of the data is compensated, a threshold of $f_{vol}/(f_{vol}+f_{db}+f_{odd})$ was set in this class, and the result was optimized by majority vote.

**Figure 11:**
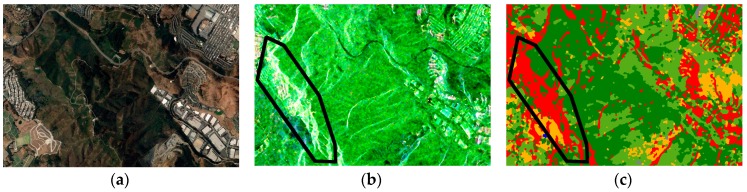

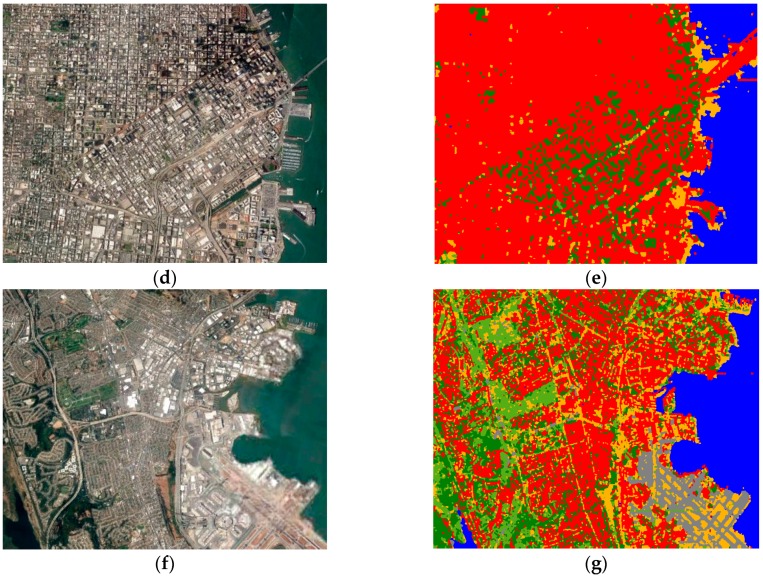
(**a**) Google Earth image of the mountain; (**b**) the corresponding Pauli-basis image; and (**c**) the corresponding classification result; (**d**) Google Earth image of oriented buildings where the intensity of the horizontal vertical channel was high; and (**e**) the corresponding classification result. (**f**) Google Earth image of the urban area around the airport; and (**g**) the corresponding classification result.

To evaluate the overall classification performance, we compared Figure 10d and the optical image. Figure 11 demonstrates a series of comparisons. As shown in the region marked with black boundary in Figure 11b,c, the misclassifications mainly occurred at the layovers of the mountain where the samples were not taken, and the high intensity caused the classifier misjudges the pixels as buildings. Slopes would also exert some effects on the polarimetric characteristics. These areas should be eliminated by digital elevation map (DEM) information in the real-world project. Additionally, as demonstrated by Figure 11d–g, some misclassifications still occurred between the trees and π/4 oriented buildings.

## 4. Conclusions

Polarimetric SAR has the potential to classify ground objects. In this paper, we demonstrated a method to mark the labels on the polarimetric SAR image. Some experiments were performed in which ground targets were roughly classified into Artificial architecture, Water body, Tree architecture, Smooth road, Bare soil, and Herbaceous vegetation. Based on Wishart distribution, a CFAR edge detector was applied to generate super-pixels more quickly and with a low adjustment cost. Then, we optimized the initial classification result through majority vote, which improved the classification accuracy, so the results better meet the mapping demand. The result shows the effectiveness of both the proposed procedure and the ability of GF-3 for land cover classification.

In this study, some classification methods were also summarized. To achieve high classification performance, a certain degree of supervision is necessary. In coarse classification, samples are easily acquired combined with the optical image, which results in rough first-level classification. Because the satellite conditions are relatively stable, and SAR images are not affected by weather and light, the classifier can be widely applied to other data. However, polarimetric SAR should have far more applications. Based on the first-level classification product, as discussed in Section 1, more fine-grained second-level classification products can be generated automatically by polarimetric decomposition in areas where samples are hard to obtain with an unknown number of categories. As Lee et al. [37,38] and Newmann et al. [39] stated, more detailed polarimetric SAR classification products for vegetation, forest, and crops can be generated. We look forward to more data and classification products for use with GF-3, which will significantly contribute to land cover surveys.

## Figures and Tables

**Table 1:** Features and physical explanation.

Feature	Physical Meaning
log(1+span1)	Describe the scattering power, which is effective in the discrimination among water, buildings, and roads.
$T_{11}/span$	Describe the target’s surface scattering property, which is effective in the extraction of bare soil and ocean water.
$T_{22}/span$	Describe the target’s double scattering property, which is effective for buildings parallel to track.
$\left|T_{23}\right|/\sqrt{T_{22}\cdot T_{33}}$	Describe the target’s reflection asymmetry, which is effective in oriented building extraction.
$\left|T_{12}\right|/span,\left|T_{23}\right|/span,\left|T_{13}\right|/span$ (selected)	Take advantage of all the information in the T.
$H,A,\underline{\alpha },RVI$	Describes the statistical characteristics of the target, which are generally used in the fine-grained discrimination of crops.
GLCM features (selected)	Describes the texture of the target, which is effective in building extraction.

**Table 2:** (**A**) shows the classification performance of the Extreme Learning Machine (ELM) and Random Forest (RF); (**B**) exhibits the proportion of selected samples, where the proportion of road samples is low; (**C**,**D**) show the evaluation of the classification results obtained with ELM and RF in the testing set of selected samples.

**(A) Classification Comparison in Selected Samples**																
**%**				**Training Set Accuracy**			**Testing Set Accuracy**				**Training Time**			**Classification Time (4500×3000)**		
ELM (100 hidden neurons, sigmoid)				80.80%			81.14%				**1.93 s**			**27.06 s**		
RF (100 trees)				**99.99%**			**95.69%**				27.8 s			181.6 s		
(**B**) **Proportion of Selected Samples**																
		**Building**				**Soil**			**Water Body**			**Tree**			**Road**	
Sample number		30,389				15,351			46,931			28,399			2763	
Proportion		24.5%				12.4%			37.9%			22.9%			2.2%	
(**C**) **Confusion Matrix by** **ELM** **in** **the Test Set of** **Selected Samples**																
**%**	**Building**		**Soil**		**Water Body**			**Tree**		**Road**			**Producer Accuracy**			**Kappa**
Building	6525		255		12			2523		1			69.38%			0.7011
Soil	97		3233		102			1104		69			70.21%			
Water body	0		29		14,011			0		39			99.52%			
Tree	2438		782		2			5288		9			62.07%			
Road	0		483		105			1		239			28.86%			
User Accuracy	71.39%		67.61%		98.45%			59.31%		66.95%			78.33%			
(**D**) **Confusion Matrix by** **RF** **in** **the Test Set of** **Selected Samples**																
**%**	**Building**		**Soil**		**Water Body**			**Tree**		**Road**			**Producer Accuracy**			**Kappa**
Building	6531		65		1			2519		0			71.64%			0.7696
Soil	56		4059		16			461		13			88.14%			
Water body	0		9		13,986			0		84			99.34%			
Tree	2370		709		0			5540		0			65.03%			
Road	0		9		30			0		791			95.53%			
User Accuracy	73.74%		83.71%		99.67%			65.02%		89.08%			83.20%			

**Table 3:** Comparison of the running time and adjusting time between simple linear iterative clustering (SLIC) and the proposed method presented in Table 3. The cost time is based on the whole polarimetric SAR image with 4500 × 3000 pixels.

%	Running Time	Adjusting Time
SLIC	8515.01 s	Running time
Proposed Method	169.03 s	8.23 s average
